# Blocking secretion of exosomes by GW4869 dampens CD8^+^ T cell exhaustion and prostate cancer progression

**DOI:** 10.1007/s13577-025-01257-0

**Published:** 2025-07-18

**Authors:** Jing Liu, Hongyan Guo, Shiqi Liu, Yinying Hu, Yanqin Huang, Jiping Rong, Fang Yuan, Ruping Wang, Zhigang Wang

**Affiliations:** 1 Jiangxi Academy of Medical Sciences, No. 461, Bayi Avenue, Nanchang, China; 2 Medical College of Nanchang University, Nanchang, China

**Keywords:** Exosome, CD8^+^ T cell exhaustion, GW4869, Prostate cancer

## Abstract

Functional exhaustion of T lymphocytes is considered an important factor in the failure of tumor treatment. Accumulating evidence has shown that tumor cells cultivate their immune microenvironment through secreting exosomes. However, the mechanism through which tumor cell-derived exosomes participate in the regulation of lymphocyte function remains unclear. In this study, we found that exosomes derived from prostate cancer (PCa) cells were able to upregulate the expression of PD-1 and TIM-3 in CD8^+^ T cells, inducing the secretion of cytokines related to T cell exhaustion and significantly decreasing the ability to kill PCa cells. Importantly, our data indicated that treatment with GW4869 could reverse the effects of PCa-derived exosomes on CD8^+^ T cells and further inhibit the growth of PCa cells in vivo and in vitro by blocking the generation of exosomes. Our findings support the notion that exosomes derived from PCa cells can induce T cell exhaustion and promote PCa progression, while treatment of GW4869 effectively rejuvenates CD8^+^ T cells and reverses the effect of PCa exosomes. These findings indicate that GW4869 has potential in the treatment of PCa.

**Supplementary Information:**

The online version contains supplementary material available at 10.1007/s13577-025-01257-0.

## Introduction

Prostate cancer (PCa) is a common male malignancy worldwide [1, 2]. Currently, despite progress in diagnosis and local therapy, the treatment of advanced metastatic castration-resistant PCa (mCRPC) is not satisfactory. Several studies have suggested that T cell exhaustion is an important factor that leads to poor clinical treatment effects in patients with advanced PCa [3–5]. However, knowledge of the regulatory mechanism underlying T cell exhaustion in PCa remains limited [6].

The continuous stimulation of tumor antigens in the tumor microenvironment (TME) causes T lymphocytes to not only gradually express multiple immunosuppressive receptors (IRs) but also decrease the secretion of effector cytokines, leading to a decline in T cell function, known as T cell exhaustion [7, 8]. Exhausted T cells are observed to be widely distributed in the tumor tissue, which allows tumor cells to escape from immunosurveillance, CD8^+^ T cell exhaustion is considered a significant contributor to the failure of tumor immunotherapy [9, 10]. Several lines of evidence have indicated that T cell exhaustion is associated with multiple mechanisms [11], including cytokines (e.g., IL-2 [12], TGF-β [13], and IL-10 [14]), inhibitory receptor signaling pathway (e.g., PD1/PD-L1 [15], TIM-3 [16], and PI3K/Akt/mTOR [17]), long-term metabolite exposure [18], or FGF2 [19]. In the TME, cancer cells can affect the immune function of CD8^+^ T cells either by directly targeting T cells or by secreting soluble factors, thus promoting the malignant transformation of various cacers [20].

Recently, exosomes—extracellular vesicles secreted by tumor cells—have been considered to possess the antigenic properties of the original tumor and can replace tumor cells to cultivate their immune microenvironment [21–23]. Exosomes are internalized by target cells either through direct fusion with the cell membrane or by attaching to specific surface proteins or receptors. The pathways include clathrin/caveolin-mediated endocytosis, lipid raft-mediated internalization, macropinocytosis, and phagocytosis. Subsequently, the contents of the exosomes are released into the cytoplasm, affecting the physiological processes of the target cells. [24, 25]. For example, a previous study [26] demonstrated that melanoma exosomes could directly impair the ability of CD14 monocytes to differentiate into functional dendritic cells (DCs), thereby inhibiting antigen presentation and subsequent T cell responses. Another study [27] also confirmed that exosomes derived from PCa cells could inhibit tumor antigen cross-presentation on DCs by inducing CD73 expression, thereby impairing the immune function of CD8^+^ T cells. In addition, exosomal miR-519e-5p was shown to be transported to CD8 + T cells to assist in tumor immune escape and distant metastasis of papillary thyroid carcinomas via downregulating NOTCH2 [28].

Together, these studies revealed a correlation between tumor exosomes and T cell exhaustion; however, the direct effect and related mechanisms of PCa exosomes on the function of CD8 + T cells are currently unclear. Specifically, the role the exosome inhibitor GW4869 plays in T cell rejuvenation has not received the attention it deserves. In this study, we confirm that exosomes from PCa cells induce T cell exhaustion. Furthermore, we show that treatment with GW4869 effectively rejuvenates CD8^+^ T cells and reverses the effect of PCa exosomes.

## Materials and methods

### Cell culture

Human PCa cell line PC-3 and mouse PCa cell line RM-1 were both purchased from the American Type Culture Collection (ATCC, Maryland, USA). PC-3 and RM-1 cells were cultured in RPMI-1640 or DMEM high glucose medium, respectively, containing 10% exosome-free FBS. Once PC-3 cells were 70–80% confluent, 10 μM GW4869 (MedChemExpress, New Jersey, USA) was added to the culture, and the cells were incubated for 48 h. After that, 100 mL of the GW4869-treated PC3 cell supernatant was collected, centrifuged at 400 × g for 10 min to remove the cellular precipitates, filtered through a 0.22 μm filter, and finally designated as CM-GW4869 for future experiments.

### Enrichment of CD8^+^ T cells

Human peripheral blood mononuclear cells (PBMCs) were isolated from the whole blood of healthy male donors using Ficoll (Solarbio Life Sciences, Beijing, China). Subsequently, CD8^+^ T cells were purified from PBMCs using CD8 magnetic beads (Miltenyi Biotec, Köln, Germany) and cultured in GT-T551 H3 media (Takara, Kyoto, Japan) supplemented with 10% fetal bovine serum, 1% penicillin–streptomycin, 5 µg/mL anti-CD3, 2 µg/mL anti-CD28, and 20 ng/mL IL-2 (Biolegend, San Diego, USA) at 37 °C with 5% CO_2_.

### Extraction of exosomes

After filtration, 100 mL of the cell supernatant was added to a Millipore ultrafiltration tube (Amicon Ultra-15, Millipore, USA) and centrifuged at 6400 × g for 40 min at 4 °C to collect the crude exosome extract. These crude exosome extracts were then purified using MagCapture™ Exosome Isolation Kit PS (Fujifilm Wako, Osaka, Japan) according to the manufacturer’s instructions. The concentration of the exosomes was determined by a bicinchoninic acid (BCA) kit, and then the exosomes were stored at − 80 °C for future use.

### Transmission electron microscopy

The exosome samples were resuspended in 20–30 μL of PBS. Then 10 μL of the exosome samples was added on a copper grid for 1 min, and 10 μL of 1% phosphotungstic acid staining solution was added dropwise on the copper net for 5 min. After the excess dye was removed, the sample was dried and observed on a transmission electron microscope.

### Nanoparticle tracking analysis

An appropriate amount of PCa exosomes was resuspended and diluted with PBS. Subsequently, the diameter of these exosomes was determined using a Zetasizer Nano ZS9003030810 analyzer (Malvern Panalytical, Malvern, UK).

### Immunofluorescence staining

Exosomes were incubated with diluted PKH67 solution (Merck, Darmstadt, Germany) at room temperature in the dark for 30 min, and then the exosomes were re-extracted by Millipore ultrafiltration. Following filtration through a 0.22 μm filter, the labeled exosomes were incubated with CD8^+^ T cells. The CD8^+^ T cells absorbing labeled exosomes were photographed on a fluorescence microscope at 6, 12, 24, and 48 h.

### Western blotting

The exosomes of each group were lysed on ice for 30 min with RIPA lysis solution containing phenylmethanesulfonyl fluoride (PMSF), proteinase inhibitors, and phosphatase inhibitors (100:1:1:1). The total protein was extracted by centrifugation, and the protein concentration was detected by a BCA assay. Then 20 μg of the total protein lysate was resolved on a 10% SDS-PAGE gel and transferred to a PVDF membrane. After blocking with 5% BSA for 2 h, the membranes were incubated with either CD63 or TSG101 antibodies (Abcam, Cambridge, UK), at a ratio of 1:1000 at 4 °C overnight. After washing with TBST three times, the corresponding secondary antibody was incubated for 2 h, and then the target protein was detected using ECL chemiluminescence.

### Cell proliferation assay

Human CD8^+^ T cells were collected and co-incubated with 5 nmol/L of carboxyfluorescein succinimidyl ester (CFSE) (eBioscience, California, USA) for 10 min at 37 °C in the dark. The labeling was then terminated by adding an equal volume of pre-cooled FBS for 10 min. The cells were washed three times, seeded onto a 24-well plate, and then incubated with GT-T551 H3 media, PCa-derived exosomes, or CM-4869 for 72 h. The control group only contains GT-T551 H3 media. Finally, the proliferative ability of the CD8^+^ T cells was determined by flow cytometry (Beckman Coulter, California, USA).

### Apoptosis assay

CD8^+^ T cells from each group were collected via centrifugation at 300 × g for 5 min at 4 °C and then washed twice with pre-cooled PBS. Subsequently, cells were resuspended with 1 × binding buffer and transferred to flow cytometry tubes. Then 5 μL of Annexin V-FITC and 10 μL of PI staining solution were incubated with the CD8^+^ T cells at room temperature for 15 min. The number of apoptotic CD8^+^ T cells was detected by flow cytometry.

### In vitro cytotoxicity assay

Human PC-3 cells (100 μL; 5 × 10^4^ cells/mL) were seeded into a 96-well plate, and co-cultured with CD8^+^ T cells treated with or without PCa exosomes (PCa-exos) for 72 h. Then, 100 μL of serum-free RPMI-1640 medium containing 10 μL of CCK-8 (MedChemExpress, New Jersey, USA) solution was added to each well, and the cells were cultured in an incubator for 4 h. The absorbance values of each well were determined at 450 nm using a microplate reader.

### Enzyme-linked immunosorbent assay (ELISA)

ELISA kits (Multisciences Biotech, Hangzhou, China) were used to measure the concentration of specific cytokines in each group, according to the manufacturer’s instructions. Briefly, the OD value of each well was determined using a microplate reader (Molecular Devices, California, USA), and the concentration of cytokines was calculated by comparing their OD value with the corresponding standard curve.

### Flow cytometry analysis

Human CD8^+^ T cells from each group were washed with PBS, and re-suspended in 200 μL of PBS. The cells were then incubated with fluorochrome-labeled antibodies (i.e., anti-CD3, anti-CD8, anti-PD-1, anti-TIM-3 antibodies) (eBioscience) for 30 min at 4 °C. After washing twice with 200 μL of PBS, the expression of the target molecules on CD8^+^ T cells was analyzed by flow cytometry.

### Subcutaneous tumorigenesis assay

Male BALB/c mice (4 weeks old) were purchased from Changsha Laboratory Animal Co.,Ltd. (Changsha, Hunan, China) and bred in the Laboratory Animal Resources at the Jiangxi Academy of Medical Sciences (Nanchang, Jiangxi, China). All animal experiments were conducted in compliance with the guidelines for handling animal experimentation-based research in China and the ARRIVE guidelines. In brief, 2 × 10^6^ RM-1 cells were mixed with either 10 μM GW4869 or 100 μg of RM-1-exos, and then injected subcutaneously into the right flanks of mice. Then 10 μM of GW4869 or 100 μg of RM-1-exos was injected subcutaneously every two days into the mice. Twenty-eight later, the mice were euthanized. The tumor volume in the mice was calculated using the following equation: tumor volume (mm^3^) = shorter diameter^2^ × longer diameter/2. The tumor tissue was then prepared into a single-cell suspension, and CD8^+^ T cells were isolated using CD8 magnetic beads. The expression of PD-1 and TIM-3 on CD8^+^ T cells was detected by flow cytometry.

### Statistical analysis

Statistical analyses were performed using GraphPad Prism 5.0. The measurement data were expressed as the mean ± standard deviation, and a *t* test was used for comparison between the two groups with normal distributions and overall variance. *P* < 0.05 was used to indicate statistically significant differences.

## Results

### Characteristics of exosomes derived from PCa cells

**Fig. 1:**
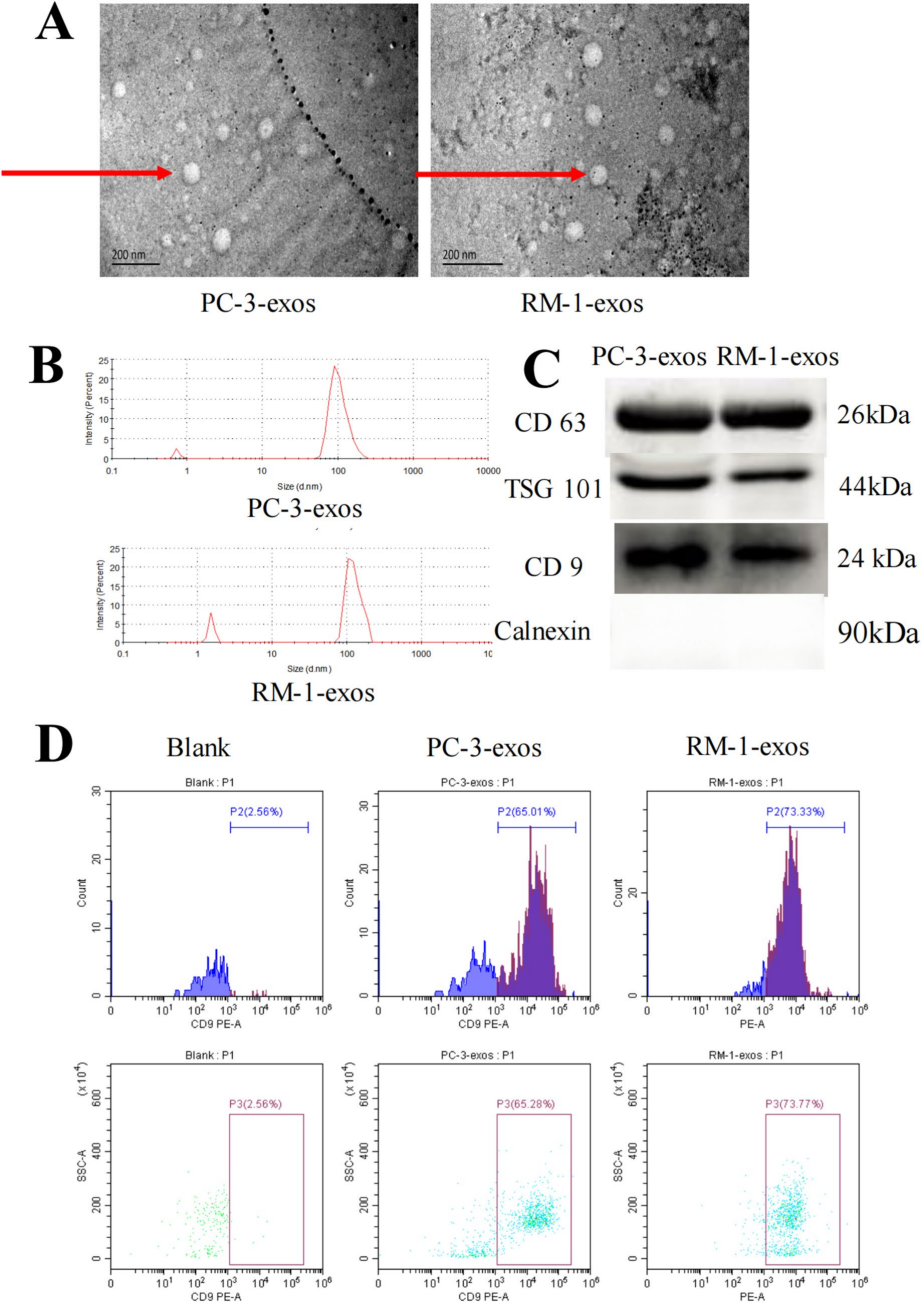
Characteristics of PCa exosomes. Human PCa cell lines PC-3 and mouse RM-1 were cultured in exosome-free media for 24 h. Subsequently, exosomes were isolated and purified from the CM of the above cultured cells using ultrafiltration and the phosphatidylserine affinity method. **A.** Electron microscopy images of exosomes derived from PC-3 or RM-1 cells. **B.** Particle size analysis of PCa exosomes. After isolation and purification, the diameter of exosomes derived from PCa cells was detected by a nanoparticle size analyzer. **C.** Analysis of the expression of TSG101, CD9, calnexin, and CD63 on PCa exosomes by western blot. **D.** CD9 expression on exosome membranes was determined by flow cytometry

Exosomes are an important means for tumor cells to communicate with other cell types in the tumor microenvironment. Through Millipore ultrafiltration and Magcapture™ Exosome Isolation Kit PS, we obtained a number of extracellular vesicles from the supernatant culture media of PC-3 or RM-1 cells. The characteristics of these extracellular vesicles were further analyzed by transmission electron microscopy, nanoparticle tracking analysis, and western blotting. As we expected, we found that these extracellular vesicles exhibit features of exosomes, including a single, circular vesicle with well-defined edges, diameter of approximately 100 nm, and expression of specific exosome markers CD63, TSG101, and CD9 (Fig. 1).

### CD8^+^ T cells efficiently absorbed PCa exosomes

**Fig. 2:**
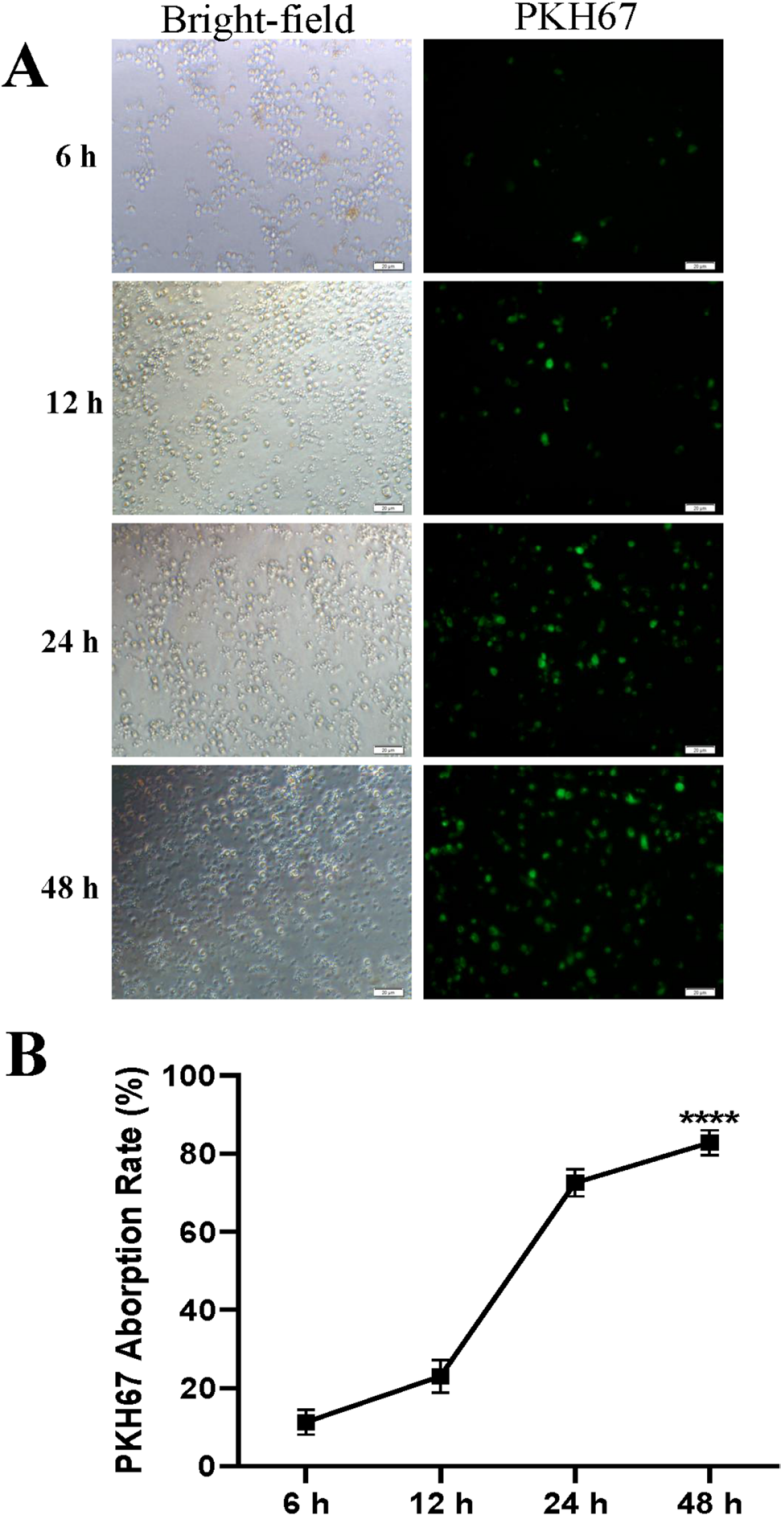
Effect of T cells absorbing PC-3-exosomes. **A**. Immunofluorescence images of CD8^+^ T cells absorbing PKH76-labeled PCa-exos. After PC-3-exos were labeled with PKH67 (green), they were added to CD8^+^ T cells and co-cultured for 6, 12, 24, and 48 h. Then, the rate of CD8^+^ T cells absorbing PC-3-exos was determined under a fluorescence microscope (original magnification, 40 ×). **B**. Quantification of CD8^+^ T cells absorbing PC-3-exos

Although several studies have shown that exosomes can transport various cargos to recipient cells, the efficiency of CD8 cells taking up tumor exosomes is still unclear. To investigate whether PC-3-exos can be taken up by CD8^+^ T cells, and thus enter CD8^+^ T cells to fulfill their biological functions, we used PKH67 green fluorescent dye to label PC-3-exos and observed uptake of exosomes by CD8^+^ T cells at 6, 12, 24, and 48 h. The results showed that after 48 h, the uptake rate of PCa exosomes by CD8^+^ T cells reached 85% (Fig. 2), indicating that PC-3-exos are able to be absorbed by CD8^+^ T cells, thereby exerting their influence on the CD8^+^ T cell 48 h later.

### PCa-exos induced T cell exhaustion in CD8^+^ T cells

**Fig. 3:**
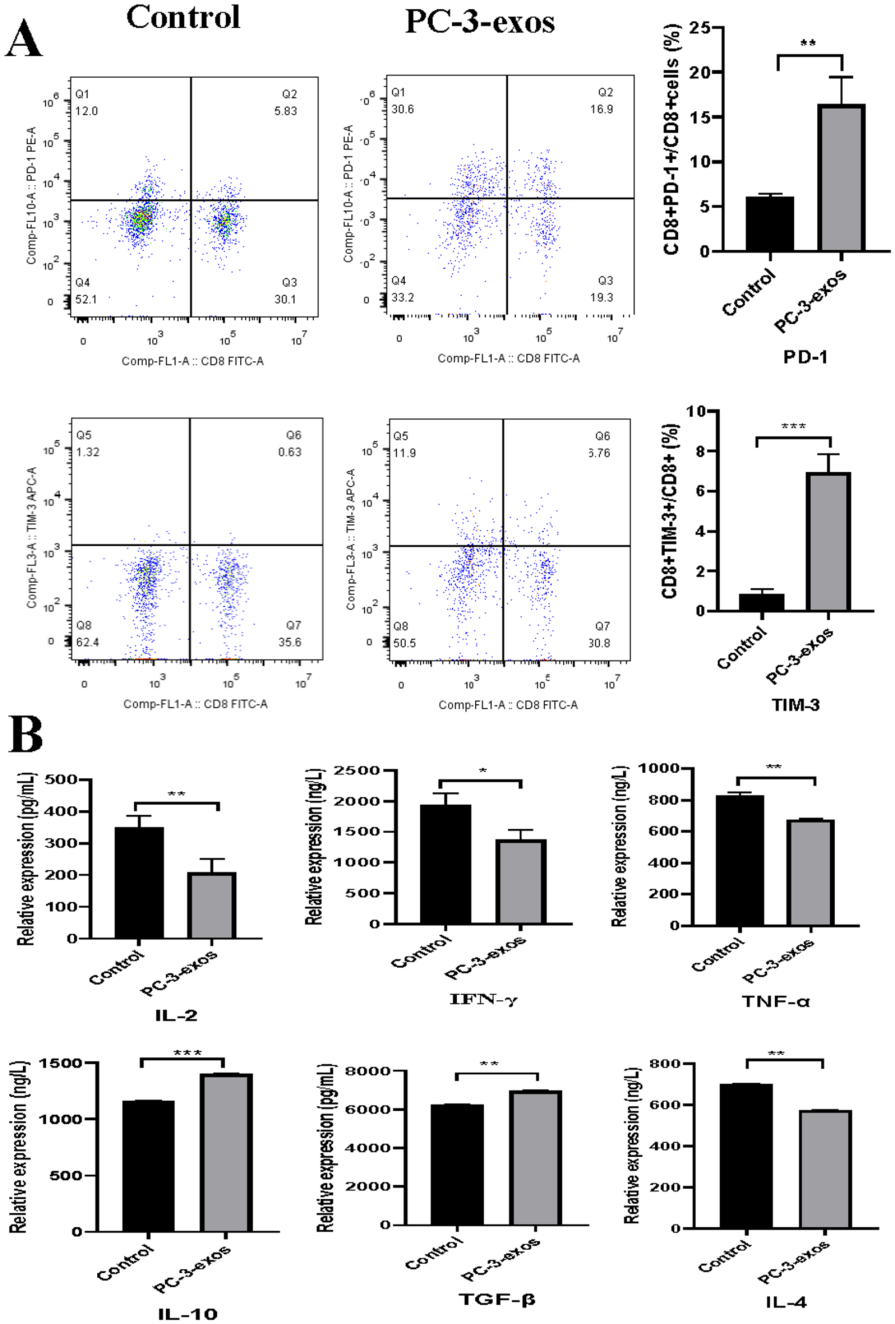
PC-3-exos induced elevated expression of IRs and cytokines related to T cell exhaustion in CD8^+^ T cells. **A**. Effects of PCa exosomes on the expression of PD-1 and TIM-3 in human CD8 + T cell. After CD8 + T cells were co-cultured with or without PC-3-exos (100 μg/mL) for 48 h, the expression of PD-1 and TIM-3 in CD8 + T cells was detected by flow cytometry; **p < 0.01, ***p < 0.001. Data represent the mean ± SEM of three independent experiments, and each experiment combines 3 replicates. **B**. The specific cytokine profile of CD8^+^ T cells induced by PC-3 exosomes. ELISA was used to measure the concentration of IL-2, IL-4, IL-10, IFN-γ, TNF-α, and TGF-β secreted by CD8 + T cells in the PC-3-exos treatment group and the control group. *p < 0.05, **p < 0.01, ***p < 0.001. Data represent the mean ± SEM of three independent experiments, and each experiment combines 3 replicates

To determine whether PCa-exos induced CD8^+^ T cell exhaustion, we examined human CD8^+^ T cells for indicators of T cell exhaustion, including the expression of IRs and secretion of specific cytokines. As shown in Fig. 3A, the expression of PD-1 and TIM-3 increased in the PC-3-exos group compared with the control group (*p* < 0.01)). Moreover, effector cytokines IL-2 (*p* < 0.05), IL-4 (*p* < 0.01), IFN-γ (*p* < 0.05), and TNF-α (*p* < 0.01) decreased in the supernatants of CD8^+^ T cells exposed to PC-3-exos, while the expression of inhibitory cytokines IL-10 (*p* < 0.001) and TGF-β (*p* < 0.01) increased (Fig. 3B). These data indicate that PC-3-exos induces a phenotype of T cell exhaustion in CD8^+^ T cells.

### PCa-exos inhibited CD8^+^ T cell growth

**Fig. 4:**
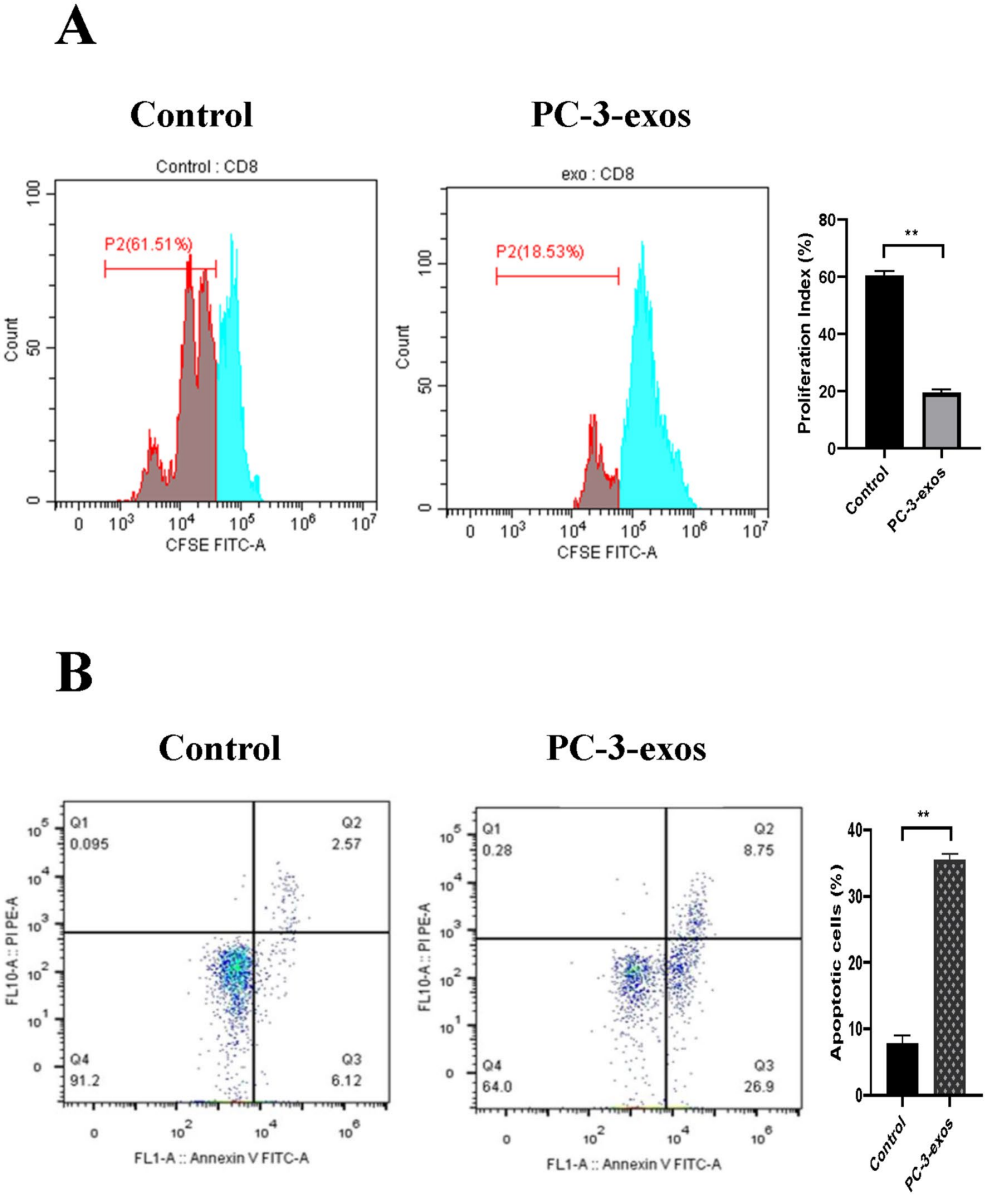
Effects of PCa exosomes on proliferation and apoptosis in human CD8^+^ T cells. **A**. PC-3 exosomes (PC-3-exos) inhibited proliferation of CD8^+^ T cells. CD8^+^ T cells isolated from human peripheral blood mononuclear cells collected from three male donors (n = 3) were labeled with CFSE and co-cultured with PC-3-exos for 3 days, and then flow cytometry was performed to assess the proliferation ability of CD8^+^ T cells treated with or without PC-3-exos; ** *p* < 0.01. Data represent the mean ± SEM of three independent experiments, and each experiment combines 3 replicates. **B**. PC-3 exosomes induced apoptosis in CD8^+^ T cells. After CD8^+^ T cells were co-cultured with or without PC-3-exos for 48 h, apoptosis levels in CD8^+^ T cells were detected in each group using the Annexin V-FITC/PI Dual-Label Apoptosis Detection Kit; ** *p* < 0.01. Data represent the mean ± SEM of three independent experiments, and each experiment combines 3 replicates

In addition to high expression of IRs and increased secretion of exhaustion-related cytokines, the decrease in the number of functional CD8^+^ T cells is also an important manifestation of T cell exhaustion. Therefore, we further investigated the effects of PCa exosomes on human CD8^+^ T cell function. Data from proliferation assays show that compared with the control group, the proliferation ability of CD8^+^ T cells in the PC-3-exos group was significantly weakened. Likewise, the number of apoptotic CD8^+^ T cells was significantly increased in the PC-3-exos group compared with the control group (*p* < 0.01) (Fig. 4). These results indicate that PC-3-exos inhibits the growth of CD8^+^ T cells.

### PCa-exos reduced the ability of CD8^+^ T cells to kill tumor cells

**Fig. 5:**
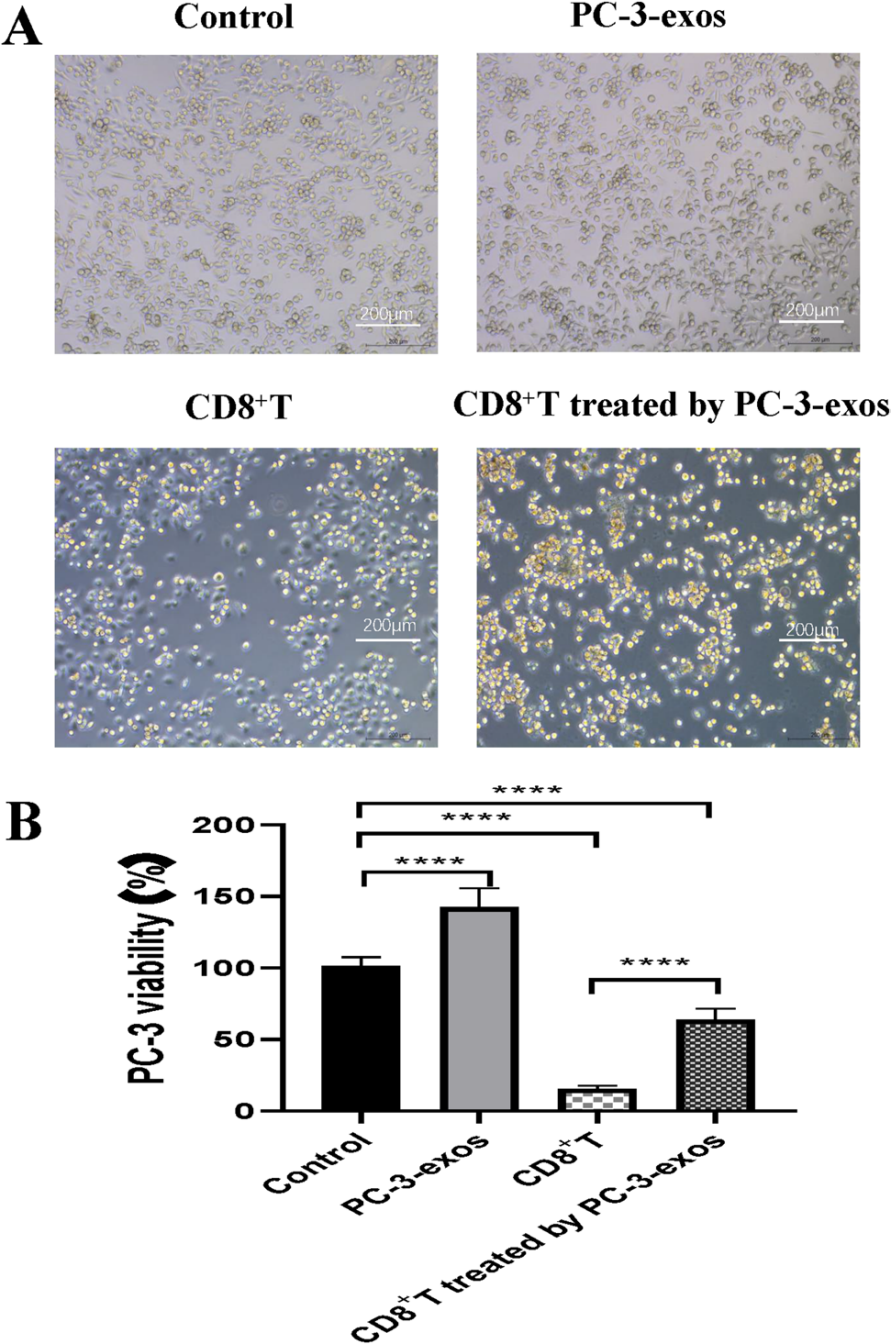
PCa exosomes reduced the ability of human CD8^+^ T cells to kill PCa cells. After treatment with or without PC-3 exosomes, CD8^+^ T cells were co-cultured with PCa cells for 72 h. The morphological changes of PC-3 cells were observed under an inverted microscope, and a CCK-8 assay was used to detect PC-3 cell viability. The experimental groups were as follows: control group 1: PBS; group 2: 100 μg/ml PC-3-exos; group 3: CD8^+^ T cells plus 100 μg/ml PC-3-exos; and group 4: CD8^+^ T cells only; *p < 0.05, **p < 0.01, ***p < 0.001, ****p < 0.0001. Data represent the mean ± SEM of three independent experiments, and each experiment combines 3 replicates. **A**. Morphology of PC-3 cells treated with different CD8^+^ T cells. **B**. Viability of PC-3 cells treated with different CD8^+^ T cells

To further clarify how PC-3-exos affects the function of human CD8^+^ T cells, we employed a CCK-8 kit to assess how exposure to PCa-exos affected the ability of CD8^+^ T cells to kill tumor cells. The experimental groups included control group (untreated PC-3 cells), PCa-exos group (PC-3 cells plus PCa-exos), CD8^+^ T cell group (PC-3 cells plus CD8^+^ T cells), and CD8^+^ T cells treated with the PC-3-exos group (PC-3 cells plus CD8^+^ T cells pre-treated with PCa-exos). The results showed that compared with the control group, the survival rate of PC-3 cells was 142.5% in the PCa-exos group, 15.56% in the CD8^+^ T cell group, and 64.18% in CD8^+^ T cells treated with the PC-3-exos group (Fig. 5). The survival rate of PC-3 cells in the CD8^+^ T cell group was significantly lower than that in CD8^+^ T cell treated by the PC-3-exos group (*p* < 0.001), indicating that PCa-exos inhibits the killing activity of CD8^+^ T cells.

### Blocking the generation of PCa exosomes with GW4869 reduced CD8^+^ T cell exhaustion and inhibited PCa growth

**Fig. 6:**
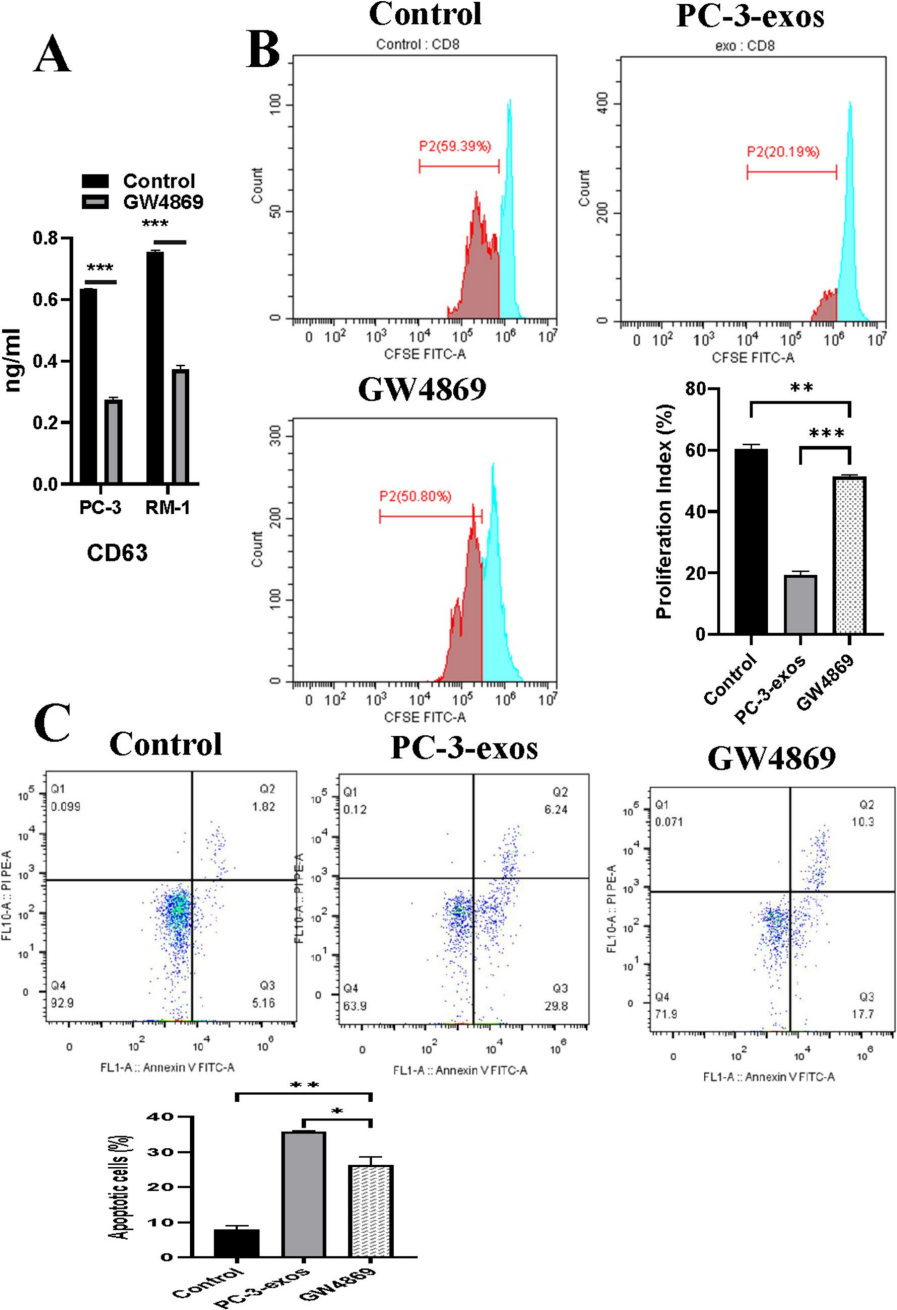
Blockade of PCa exosome release with GW4869 affected proliferation and apoptosis of human CD8^+^ T cells. **A**. Effect of GW4869 on amount of CD63 in CM. After treatment with 10 μM GW4869 for 48 h, the CM of PC-3 cells or RM-1 cells was collected and labeled as CM-GW4869-PC-3 or CM-GW4869-RM-1. Then, content of CD63 in the above CM was analyzed by ELISA. *p ≤ 0.05, **p ≤ 0.01, ***p ≤ 0.001. Data represent the mean ± SEM of three independent experiments, and each experiment combines 3 replicates. **B**. Blockade of PCa exosome release with GW4869 promoted proliferation of CD8^+^ T cells. CD8^+^ T cells were co-cultured with PCa-exos or CM-GW4869 for 3 days, and then flow cytometry was performed to assess the proliferation ability of CD8^+^ T cells in each group; ** *p* < 0.01. The experimental groups were as follows: control group: PBS; PC-3-exos group: PC-3-exos; and GW4869 group: CM-GW4869-PC-3. Data represent the mean ± SEM of three independent experiments, and each experiment combines 3 replicates. **C**. Blockade of PCa exosome release with GW4869 reduced the apoptosis levels in CD8^+^ T cells. After CD8^+^ T cells were co-cultured with PCa exosomes or CM-GW4869-PC-3 for 48 h, the apoptosis levels in CD8 + T cells in each group were determined by the Annexin V-FITC/PI Dual-Label Apoptosis Detection Kit; ** *p* < 0.01. The experimental groups were as follows: control group: PBS; PC-3-exos group: PC-3-exos; and GW4869 group: CM-PC-3-GW4869. Data represent the mean ± SEM of three independent experiments, and each experiment combines 3 replicates

**Fig. 7:**
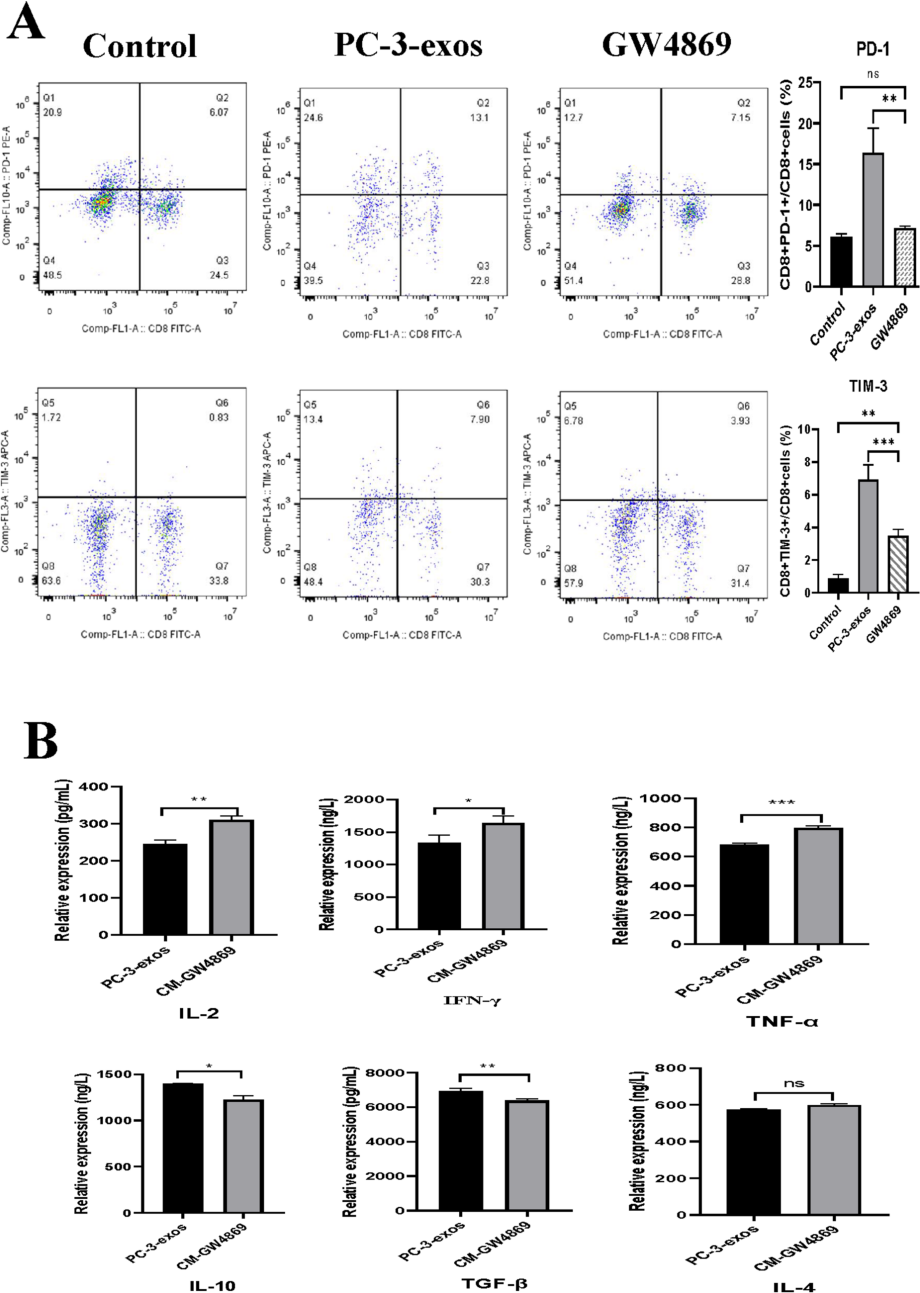
Blockade of PCa exosome release reversed CD8^+^ T cell exhaustion induced by exosomes. **A**. Blockade of PCa exosome release with GW4869 downregulated expression of PD-1 and TIM-3 on CD8^+^ T cells. After human CD8^+^ T cells were co-cultured with PCa exosomes or CM-GW4869-PC-3 for 48 h, expression of PD-1 and TIM-3 on CD8^+^ T cell was determined by flow cytometry; **p < 0.01. Data represent the mean ± SEM of three independent experiments, and each experiment combines 3 replicates. The experimental groups were as follows: control group: PBS; PC-3-exos group: PC-3-exos; and GW4869 group: CM-GW4869-PC-3. **B**. Effects of blocking PCa exosome release with GW4869 on cytokine secretion in human CD8^+^ T cells. After CD8.^+^ T cells were co-cultured PCa exosomes or CM-GW4869-PC-3 for 48 h, CM from each group was collected and analyzed the concentration of IL-2, IL-4, IL-10, IFN-γ, TNF-α, and TGF-β by ELISA. The experimental groups were as follows: PC-3-exos group: PC-3-exos; GW4869 group: CM-GW4869-PC-3. Data represent the mean ± SEM of three independent experiments, and each experiment combines 3 replicates. ns: *p* > 0.05, *p < 0.05, **p < 0.01, ***p < 0.001

**Fig. 8:**
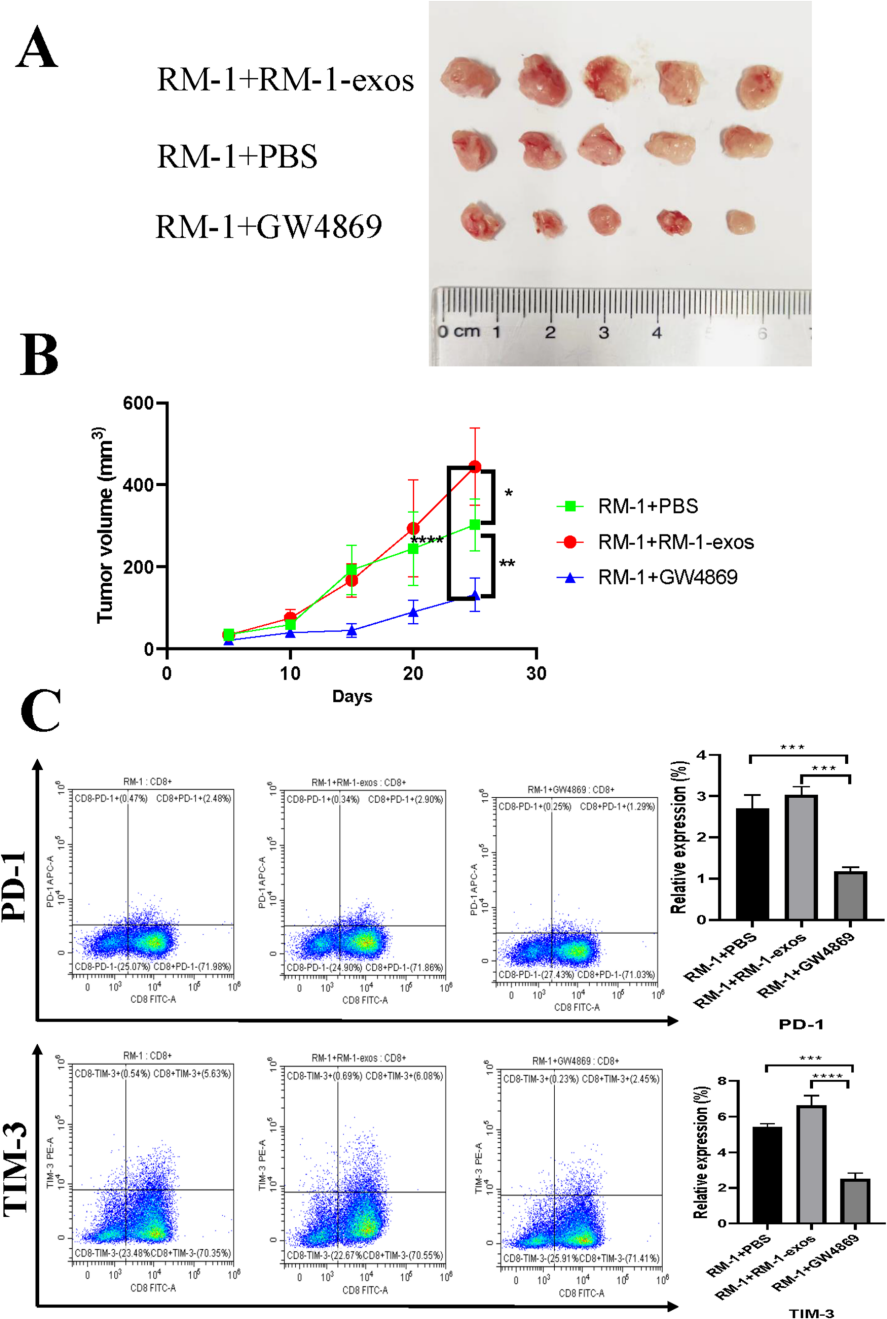
PCa exosomes induced CD8^+^ T cell exhaustion and inhibited tumor growth in vivo. **A**. Effects of RM-1-exos and GW4869 on prostate cancer tumor growth in tumor xenograft mice (n = 5). After the subcutaneous injection of RM-1 cells (2 × 10^6^) treated with GW4869 or RM-1-exos or PBS in male BALB/c mice, tumor volume was measured every 2 days. **B**. Tumor growth curves of the mice. **C**. The expression of PD-1 and TIM-3 on the surface of tumor-infiltrating CD8^+^ T cells was detected by flow cytometry in each group. After tumor formation, tumor-infiltrating CD8.^+^ T cells were sorted using immunology magnetic beads, and the expression of PD-1 and TIM-3 was analyzed using flow cytometry. ** *p* < 0.01, *** *p* < 0.001, **** *p* < 0.0001

As an inhibitor of neutral sphingomyelinase, GW4869 is widely used to block exosome production [29]. To evaluate if GW4869 could reverse the effects of PCa exosomes on CD8^+^ T cells, we initially detected the amount of exosomes in the CM from PC-3 or RM-1 cells treated by GW4869, and then investigated the effect of CM-GW4869 on human CD8^+^ T cells in vitro. As indicated in Figs. 6, the amount of CD63, a key molecular marker of exosomes, decreased by 57.3% or 54.7% in CM-GW4869 from RM-1 or PC-3 cells respectively. Moreover, the proliferation ability of CD8^+^ T cells in the GW4869 group increased compared with the PC-3-exos group (*p* < 0.01), and the level of apoptotic CD8^+^ T cells in the GW4869 group (25.51%) significantly decreased compared with that of PC-3-exos group (*p* < 0.01). In addition, the expression of two IRs, PD-1 and TIM-3, showed a significant decrease in the GW4869 group, concomitant with decreased expression of IL-10 (*p* < 0.001) and TGF-β (*p* < 0.01) and increased expression of IL-2 (*p* < 0.05), IFN-γ (*p* < 0.05), and TNF-α (*p* < 0.05) (Fig. 7). Next, we constructed a mouse model of PCa with BALB/c mice injected with 1) RM-1 cells mixed with PBS or 2) RM-1 cells mixed with 10 μM GW4869, or RM-1 cells mixed with 100 μg of RM-1-exos. As shown in Fig. 8, the tumor volume from the RM-1-exos group was significantly larger than that of other group, while the tumor volume of the GW4869 group was the smallest among the three groups (RM-1 vs. GW4869, *p* < 0.001; RM-1-exos vs. GW4869, *p* < 0.0001). In addition, we detected the expression of PD-1 and TIM-3 on mouse CD8^+^ T cells from each group by flow cytometry. The result showed that the expression of PD-1 and TIM-3 both was higher on the surface of the tumor-infiltrating CD8^+^ T cells from the RM-1-exos group than that from the other groups. These results suggest that blocking the generation of PCa exosomes with GW4869 reduced CD8 + T cell exhaustion and inhibited PCa growth in vivo.

## Discussion

In this study, we demonstrate that exosomes derived from PCa cells contribute to CD8^+^ T cell exhaustion via upregulating PD-1 and TIM-3 and perturbing the secretion of effector cytokines, such as IL-2, IFN-γ, TNF-α, and TGF-β, thereby reducing the tumor-killing ability of CD8^+^ T cells. This study also demonstrated that GW4869, an inhibitor of exosome production, effectively restored the function of T cells and inhibited the growth of PCa. Thus, GW4869 has clinical potential as a future therapy for tumor treatment.

First, we obtained exosomes from the PCa cell lines, PC-3 and RM-1, through phosphatidylserine affinity chromatography and analyzed their characteristics. As shown in Fig. 1, most of the exosomes were single, circular vesicles with well-defined edges and a diameter of approximately 100 nm. We also assessed the expression of exosome-specific molecular markers, such as CD9, CD63, and TSG101, on the isolated exosomes. Our results were consistent with the exosome characteristics reported in the literature [30, 31]. We further observed the efficiency of CD8^+^ T cells to uptake the exosomes. After incubating CD8^+^ T cells with PCa exosomes for 48 h, we found, as expected, that 85% of the CD8^+^ T cells successfully absorbed PCa exosomes (Fig. 2), indicating that exosomes could influence the physiological activity of CD8^+^ T cells. These results further provide a basis for the exosomes to be used as a “carrier” to target drugs to T cells.

Exosomes from tumor cells have been demonstrated to exhibit a similar anti-immune ability as tumor cells and act as tumor antigens that are recognized by T cells, ultimately leading to T cell dysfunction and immune escape [32, 33]. As shown in Fig. 4, PC-3-exos significantly inhibited the proliferation of CD8^+^ T cells and promoted CD8^+^ T cell apoptosis. Moreover, the expression of cell surface exhaustion indicators, PD-1 and TIM-3, was significantly elevated on CD8^+^ T cells exposed to PC-3-exos. The secretion of IL-2, IL-4, TNF-α, and IFN-γ reduced in CD8^+^ T cells exposed to PC-3-exos, while the secretion of IL-10 and TGF-β increased (Fig. 3). Accumulating evidence has suggested that characteristics of T cell exhaustion include a high expression of inhibitory receptors, a specific effector cytokine secretion profile (IL-2^high^, IL-4 ^high^, TNF-α^high^, IFN-γ ^high^, IL-10 ^low^, TGF-β^low^), and severely impaired proliferation ability [34–37], our results are consistent with these reports of T cell exhaustion from other tumors [15, 38, 39].

Unfortunately, we are currently unable to confirm which molecules in the PCa exosomes are responsible for the expression changes in molecular markers related to T cell exhaustion in CD8^+^ T cells. As indicated in Fig. S1, PC-3 exosomes can up-regulate the phosphorylated STAT3 (p-STAT3) proteins, while down-regulating the expression of c-MYC in CD8^+^ T cells. Several studies have confirmed that a decline in the phosphorylation of STAT3 contributes to T cell exhaustion by directly or indirectly reducing the expression of TOX and PD-1 [40, 41], and inhibition of STAT3 abrogates the expression of Tim-3 and IL-10 in T cells [42, 43]. Moreover, the downregulation of MYC is considered an important factor leading to T cell exhaustion [44]. We speculate that PC-3 exosomes may induce CD8^+^ T cell exhaustion through the synergistic effects of multiple mechanisms. This is primarily because exosomes typically contain a variety of bioactive substances that exert various effects on CD8^+^ T cells. Therefore, we speculated that in this study, priming of T cell exhaustion by PCa-derived exosomes is possibly attributed to the synergistic effect of multiple biomolecules in exosomes, rather than a single molecule or mechanism. We performed subsequent experiments to further demonstrate that exposure of CD8^+^ T cells to PCa-derived exosomes significantly inhibits the killing-tumor ability of CD8^+^ T cells (Fig. 5). These data indicate that exosomes derived from PCa cells are able to induce T cell exhaustion, thus promoting PCa growth.

Another important finding of this study is that GW4869 can effectively block CD8^+^ T cell exhaustion. Previous studies have shown that blocking the IR pathway [29, 45], activation of the transcription factor NFAT5 [46], or IL-7^5^ rejuvenates T cells and enhances the therapeutic effect on tumors. Recently, Peng et al. used GW4869 to block the secretion of PCa exosomes and impede macrophage M2 differentiation, thereby inhibiting PCa progression [47]. Here, we found that 10 μM GW4869 had a significant inhibitory effect on generation of PC-3 and RM-1 exosomes, up to 57% or 54% respectively (Fig. 6A), and after culturing with CM-GW4869, the proliferation rate of CD8^+^ T cells increased (Fig. 6B) and the number of apoptotic cells decreased compared with CD8^+^ T cells induced by PCa exosomes (Fig. 6C). In addition, along with the downregulation of PD-1 and TIM-3, CD8^+^ T cells from the GW4869 group increased the secretion of effector cytokines IL-2, TNF-α, and IFN-γ, and decreased the secretion of inhibitory cytokines IL-10 and TGF-β (Fig. 7). These results suggest that GW4869 could impair the ability of PCa cells promoting T cell exhaustion.

To verify this conclusion, we established a mouse subcutaneous PCa model using RM-1 cells and further explored the effects of GW4869 or PCa exosomes on T cell function and tumor growth in vivo. As shown in Fig. 8, the tumor volume of the PCa exosome group was significantly higher compared with that of the control group, and there was also a significant increase in the expression of PD-1 and TIM-3 on CD8^+^ T cells from the PCa exosome group. However, the tumor size of the GW4869 group was significantly reduced compared with the PCa exosome group, which was accompanied by a significant decrease in the expression of both PD-1 and TIM-3 on CD8^+^ T cells. Recently, several studies have indicated that GW4869 treatment can inhibit tumor growth in nude mice, potentially via regulation of the TME through blocking the secretion of tumor cell exosomes [48, 49]. Another study found that GW4869 blocked the generation or secretion of exosomes, which inhibited the differentiation of macrophages into an immunosuppressive phenotype, thereby suppressing tumor growth in a mouse subcutaneous tumor model [50]. Our results further support that PCa exosomes promote CD8^+^ T cell exhaustion, and GW4869 can effectively inhibit the induction of T cell exhaustion by PCa cells via blockade of exosome generation.

In conclusion, our findings highlight the significance of PCa-derived exosomes in educating CD8^+^ T cells. PCa-derived exosomes not only are capable of inducing CD8^+^ T cell exhaustion but may also affect PCa progression by modulating immune cells and the TME. Importantly, our study strongly supports the application of GW4869 in clinical tumor immunotherapy based on its inhibitory effect on exosome secretion. Thus, targeting the exosome generation could improve anti-tumor immunity, thereby benefiting tumor patients.

## Supplementary Information

Below is the link to the electronic supplementary material.

Supplementary file1 (DOCX 948 KB)

Supplementary file2 (DOCX 214 KB)

Supplementary file3 (DOCX 34 KB)

Supplementary file4 (DOCX 37114 KB)

## Data Availability

The data used to support the findings of this study are available from the corresponding author upon request.

## Abbreviations

PCa: Prostate cancer
PCa-exos: Exosomes from prostate cancer cells
PC-3-exo: Exosome from PC-3 cells
RM-1-exo: Exosome from RM-1 cells
CM-GW4869: Conditioned media from PC-3 cells treated with GW4869
IR: Immunosuppressive receptor
PD-1: Programmed cell death protein
TIM-3: T-cell immunoglobulin mucin 3
