# Supplementary material for: Blocking secretion of exosomes by GW4869 dampens CD8+ T cell exhaustion and prostate cancer progression
Source: Hum Cell. 2025 Jul 18;38(5):131. doi: 10.1007/s13577-025-01257-0 (PMC12274262; doi:10.1007/s13577-025-01257-0)
Supplement: Supplementary file 1 — Supplementary file1 (DOCX 948 KB) [file 13577_2025_1257_MOESM1_ESM.docx]

**S1. Expression of exosomal marker CD63,** **TSG 101, CD9 and Calnexin was determined by Western blot.**

**CD63(53kDa): Lane 1: Marker; Lane 2: PC-3 exosomes; Lane 3: RM-1 exosomes; Lane 4: Marker.**


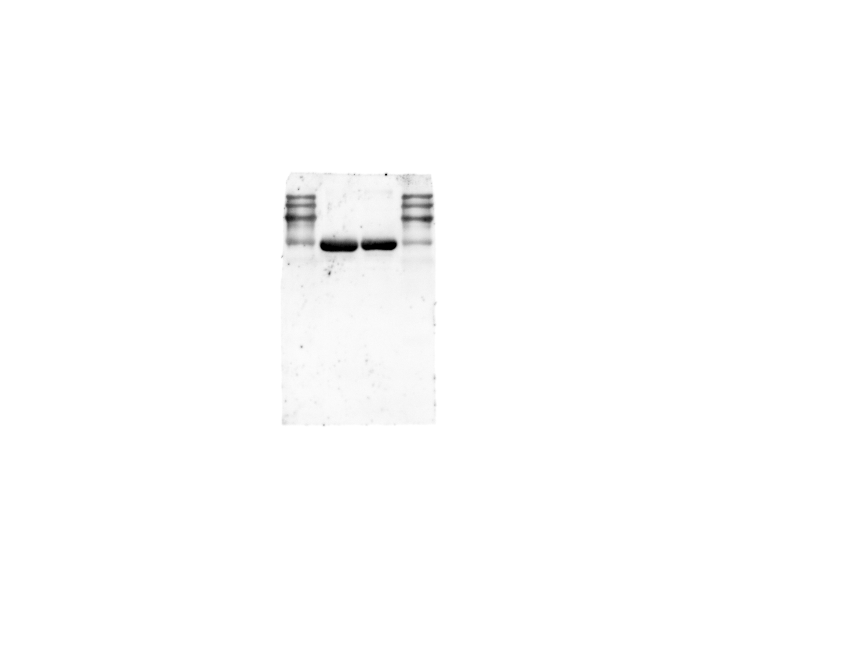


**53KDa**

**CD63**

**TSG 101 (40kDa): Lane 1: PC-3 exosomes; Lane 2: RM-1 exosomes.**

**
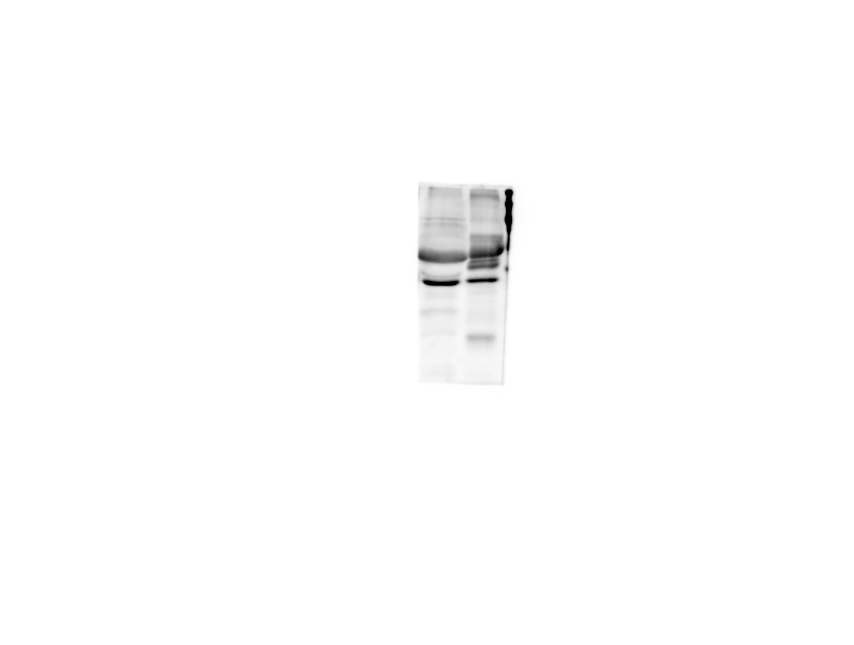
**

**TSG 101**

**40KDa**

**CD9 (24kDa):** **Lane 1: Marker; Lane 2: PC-3 exosomes; Lane 3: RM-1 exosomes;** **Lane4: Marker.**


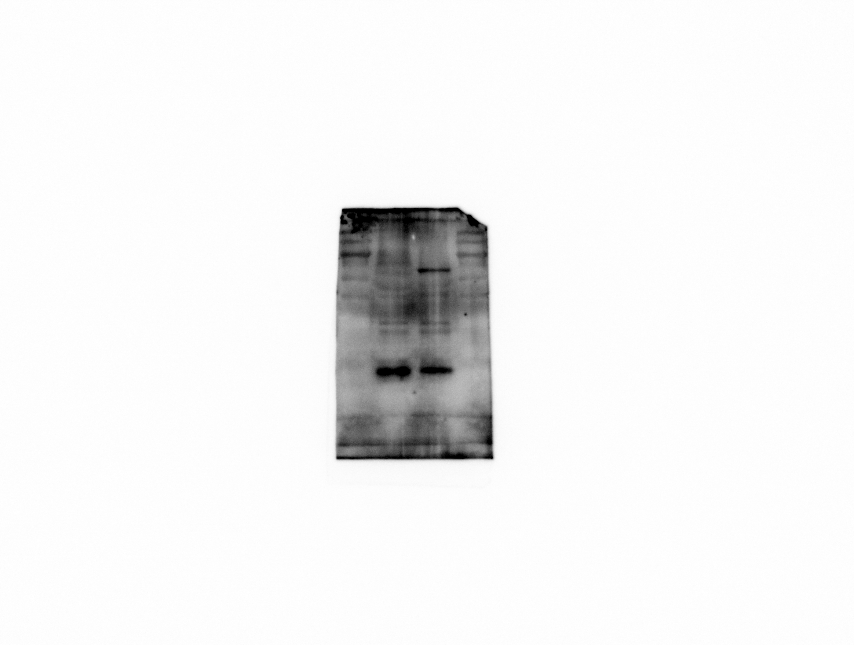


**24KDa**

**CD-9**

**Calnexin (90KDa): Lane 1: Marker; Lane 2: PC-3 cells; Lane 3: PC-3 exosomes;** **Lane4: RM-1 exosomes.**

**
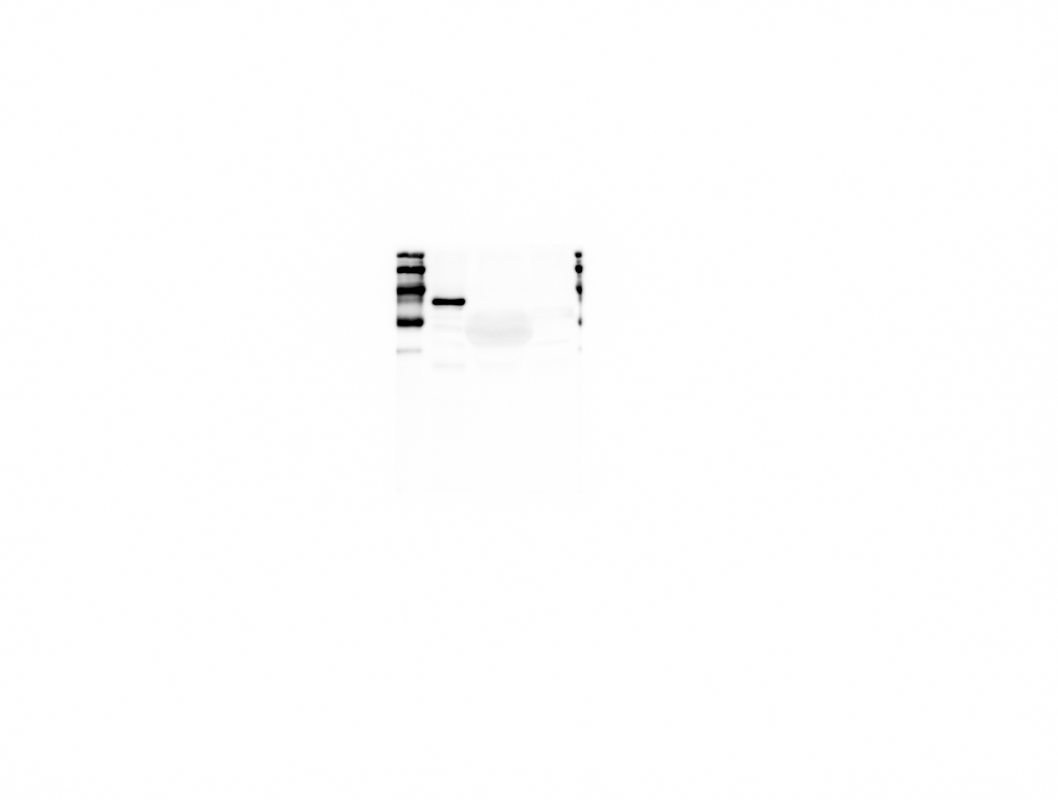
**

**Calnexin**

**90KDa**

**70KDa**

**S2.** **WB analysis of c-MYC,** **p-STAT3 and p-FOXO1 was determined by Western blot.**

**A.WB analysis of c-MYC was determined by Western blot.**

**β-actin(42kDa): Lane 1: Marker；Lane 2: Control group 1; Lane 3: PC-3 exosome group 1; Lane 4: GW4869 group 1; Lane 5: Control group 2; Lane 6: PC-3 exosome group 2; Lane 7: GW4869 group 2; Lane 8:Control group 3; Lane 9: PC-3 exosome group 3; Lane 10: GW4869 group 3.**

**
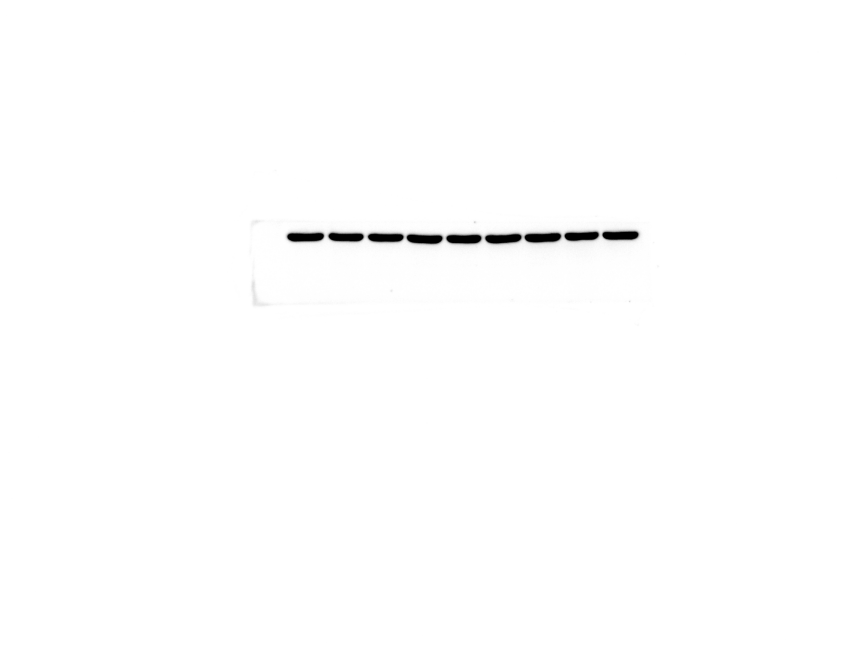
**

**42KDa**

**β-actin**

**p-FOXO1(80kDa): Lane 1: Marker；Lane 2: Control group 1; Lane 3: PC-3 exosome group 1; Lane 4: GW4869 group 1; Lane 5: Control group 2; Lane 6: PC-3 exosome group 2; Lane 7: GW4869 group 2; Lane 8:Control group 3; Lane 9: PC-3 exosome group 3; Lane 10: GW4869 group 3.**


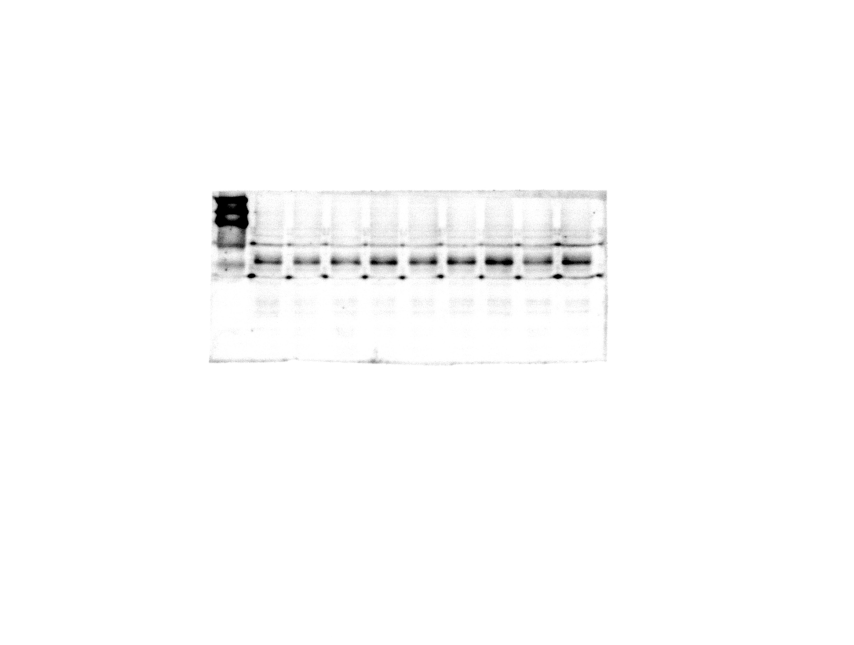


**p-c-MYC**

**62KDa**

**B.WB analysis of p-STAT3 was determined by Western blot.**

**
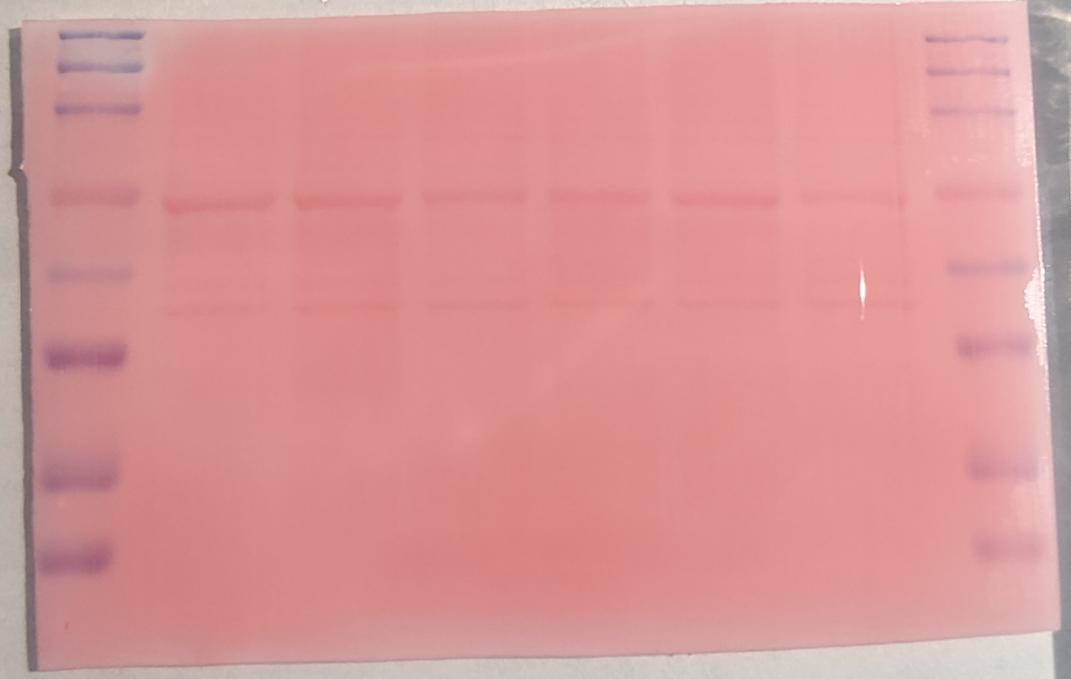
**

**55KDaa**

**70KDa**

**100KDaa**

**Ponceau S staining of the membrane after SDS-PAGE and protein transfer, uncropped, related to STAT3 of Fig.S1. Lane 1:Marker; Lane 2:Control group 1; Lane 3: PC-3 exosome group 1; Lane 4: GW4869 group 1; Lane 5: Control group 2; Lane 6: PC-3 exosome group 2; Lane 7: GW4869 group 2;Lane 8:Marker.**

**β-actin(42kDa): Lane 1:Marker; Lane 2:Control group 1; Lane 3: PC-3 exosome group 1; Lane 4: GW4869 group 1; Lane 5: Control group 2; Lane 6: PC-3 exosome group 2; Lane 7: GW4869 group 2;Lane 8:Marker.**

**
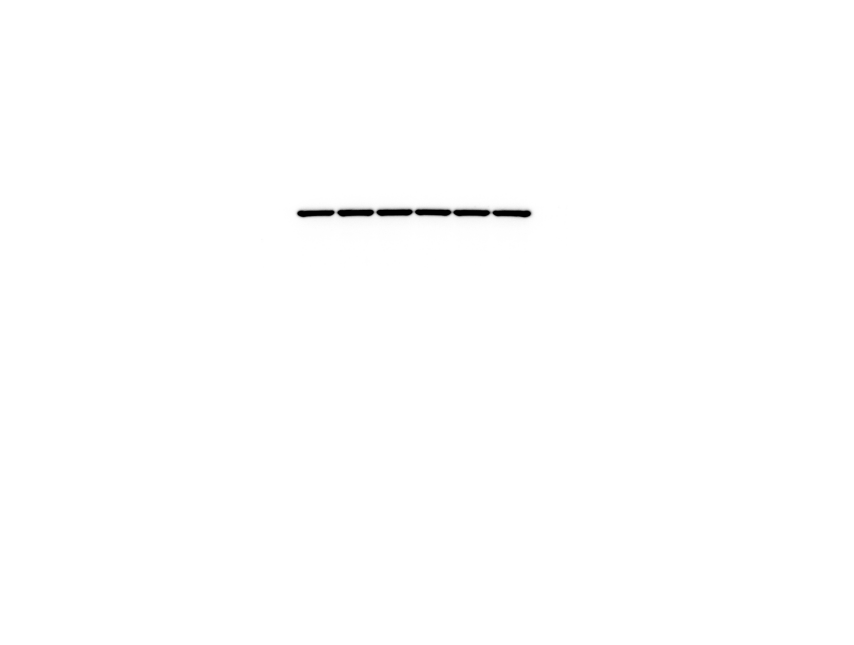
**

**42KDa**

**β-actin**

**p-STAT3(88kDa): Lane 1:Marker; Lane 2:Control group 1; Lane 3: PC-3 exosome group 1; Lane 4: GW4869 group 1; Lane 5: Control group 2; Lane 6: PC-3 exosome group 2; Lane 7: GW4869 group 2;Lane 8:Marker.**

**
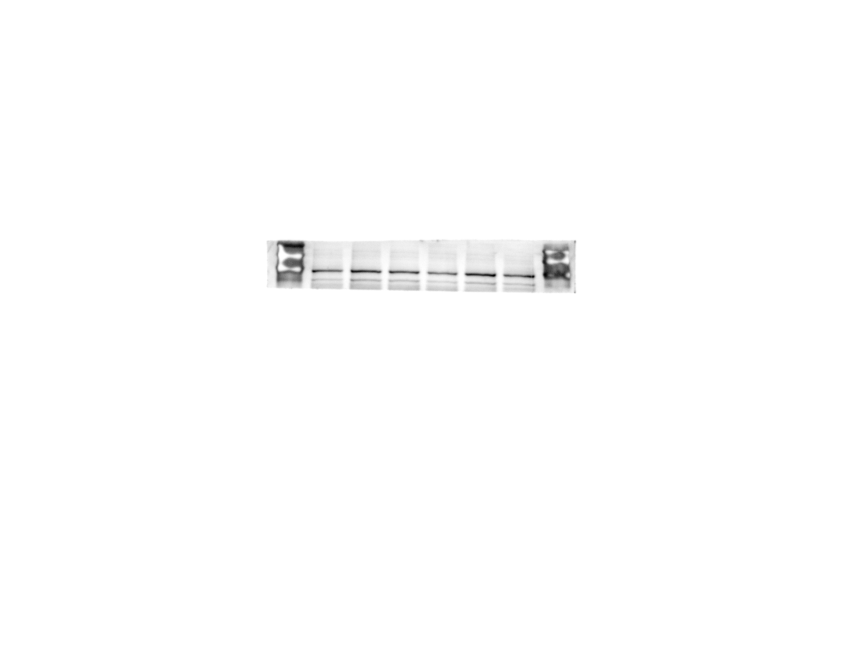
**

**88KDa**

**p-STAT3**
