# Supplementary material for: Blocking secretion of exosomes by GW4869 dampens CD8+ T cell exhaustion and prostate cancer progression
Source: Hum Cell. 2025 Jul 18;38(5):131. doi: 10.1007/s13577-025-01257-0 (PMC12274262; doi:10.1007/s13577-025-01257-0)

Figure S1. PC-3 exosomes down-regulated c-MYC, while up-regulated phosphorylated STAT3 (p-STAT3) protein in CD8+ T cells.

After treating human CD8+ T cells with PCa exosomes, CM-GW4869-PC-3, or media, total protein was isolated from these CD8+ T cells and quantified using a BCA assay. The relative protein expression of c-MYC and p-STAT3 was determined by densitometric analysis from three independent samples. Statistical significance was indicated as follows: ns, p > 0.05; *, p < 0.05; **, p < 0.01; ***, p < 0.001.


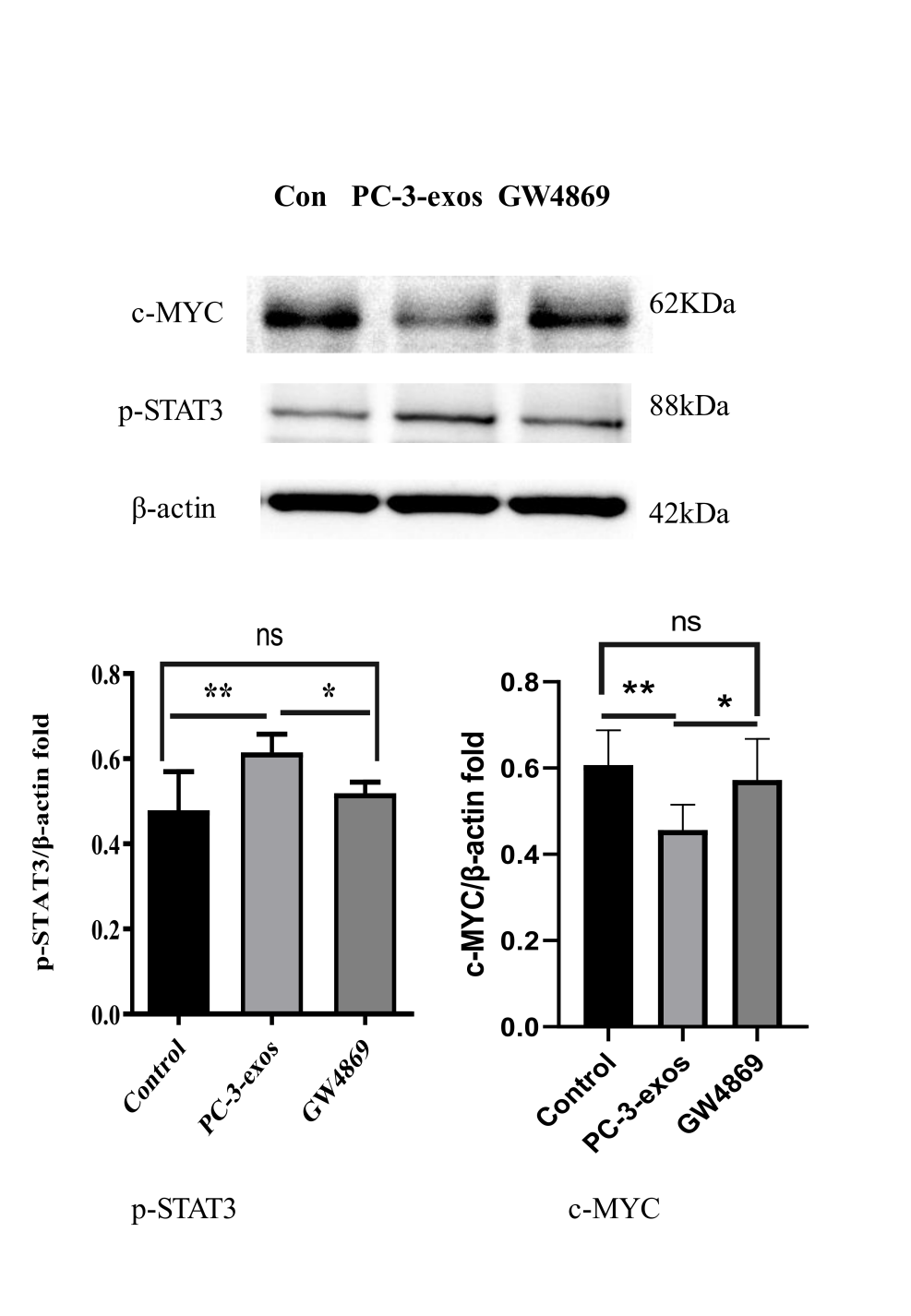

Supplement: Supplementary file 2 — Supplementary file2 (DOCX 214 KB) [file 13577_2025_1257_MOESM2_ESM.docx]
