# Supplementary material for: Blocking secretion of exosomes by GW4869 dampens CD8+ T cell exhaustion and prostate cancer progression
Source: Hum Cell. 2025 Jul 18;38(5):131. doi: 10.1007/s13577-025-01257-0 (PMC12274262; doi:10.1007/s13577-025-01257-0)

Figure S2. PC-3 exosomes significantly down-regulated the expression of perforin on CD8+ T cells.

Upon treatment of human CD8+ T cells with PCa exosomes, CM-GW4869-PC-3, or media, the expression levels of perforin and granzyme were quantified using an ELISA assay. The upper panel illustrates the differential expression of perforin across the three groups of samples, while the lower panel shows the differential expression of granzyme B. Data are presented as the mean ± SEM from three independent experiments, each consisting of 3 replicates. Statistical significance is indicated as follows: ns, p > 0.05; *, p < 0.05; **, p < 0.01; ***, p < 0.001.


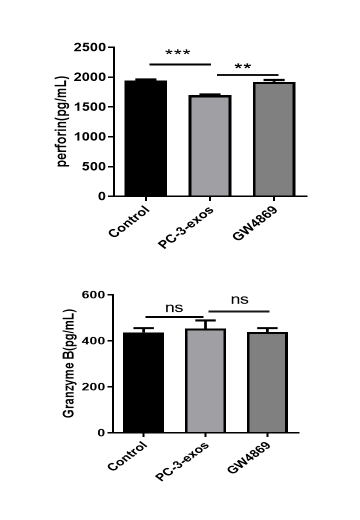

Supplement: Supplementary file 3 — Supplementary file3 (DOCX 34 KB) [file 13577_2025_1257_MOESM3_ESM.docx]
