# Supplementary material for: Blocking secretion of exosomes by GW4869 dampens CD8+ T cell exhaustion and prostate cancer progression
Source: Hum Cell. 2025 Jul 18;38(5):131. doi: 10.1007/s13577-025-01257-0 (PMC12274262; doi:10.1007/s13577-025-01257-0)
Supplement: Supplementary file 4 — Supplementary file4 (DOCX 37114 KB) [file 13577_2025_1257_MOESM4_ESM.docx]

Figure S3. PD-1 and TIM-3 were overexpressed in prostate cancer tissues compared with those in the adjacent tumor tissues in the PC xenograft model.

Immunohistochemistry was used to analyze the expression of PD-1 and TIM-3 in prostate cancer tissues from PC xenograft mice. The upper panel displays the H&E staining and immunohistochemical images, while the lower panel represents the corresponding analysis results.
